# LRRC15^+^ myofibroblasts dictate the stromal setpoint to suppress tumour immunity

**DOI:** 10.1038/s41586-022-05272-1

**Published:** 2022-09-28

**Authors:** Akshay T. Krishnamurty, Justin A. Shyer, Minh Thai, Vineela Gandham, Matthew B. Buechler, Yeqing Angela Yang, Rachana N. Pradhan, Amber W. Wang, Patricia L. Sanchez, Yan Qu, Beatrice Breart, Cécile Chalouni, Debra Dunlap, James Ziai, Justin Elstrott, Neelie Zacharias, Weiguang Mao, Rebecca K. Rowntree, Jack Sadowsky, Gail D. Lewis, Thomas H. Pillow, Barzin Y. Nabet, Romain Banchereau, Lucinda Tam, Roger Caothien, Natasha Bacarro, Merone Roose-Girma, Zora Modrusan, Sanjeev Mariathasan, Sören Müller, Shannon J. Turley

**Affiliations:** Genentech, South San Francisco, CA USA

**Keywords:** Immunosurveillance, Translational immunology, Cancer immunotherapy, Cancer microenvironment, Tumour immunology

## Abstract

Recent single-cell studies of cancer in both mice and humans have identified the emergence of a myofibroblast population specifically marked by the highly restricted leucine-rich-repeat-containing protein 15 (LRRC15)^1–3^. However, the molecular signals that underlie the development of LRRC15^+^ cancer-associated fibroblasts (CAFs) and their direct impact on anti-tumour immunity are uncharacterized. Here in mouse models of pancreatic cancer, we provide in vivo genetic evidence that TGFβ receptor type 2 signalling in healthy dermatopontin^+^ universal fibroblasts is essential for the development of cancer-associated LRRC15^+^ myofibroblasts. This axis also predominantly drives fibroblast lineage diversity in human cancers. Using newly developed *Lrrc15–*diphtheria toxin receptor knock-in mice to selectively deplete LRRC15^+^ CAFs, we show that depletion of this population markedly reduces the total tumour fibroblast content. Moreover, the CAF composition is recalibrated towards universal fibroblasts. This relieves direct suppression of tumour-infiltrating CD8^+^ T cells to enhance their effector function and augments tumour regression in response to anti-PDL1 immune checkpoint blockade. Collectively, these findings demonstrate that TGFβ-dependent LRRC15^+^ CAFs dictate the tumour-fibroblast setpoint to promote tumour growth. These cells also directly suppress CD8^+^ T cell function and limit responsiveness to checkpoint blockade. Development of treatments that restore the homeostatic fibroblast setpoint by reducing the population of pro-disease LRRC15^+^ myofibroblasts may improve patient survival and response to immunotherapy.

## Abstract (web summary)

LRRC15-positive cancer-associated fibroblasts constitute a pivotal axis in tumorigenesis and are potential therapeutic targets to improve responses to immune checkpoint blockade.

## Main

CAFs play a key part in shaping the tumour microenvironment (TME) and response to cancer immunotherapy^4–6^. Previous studies of gene expression data from tumours of patients who have received immune checkpoint blockade (ICB) therapies have inferred an association between CAF abundance and lack of response to immunotherapy^7,8^. Therapeutic agents that appropriately target CAFs may alleviate this resistance, but remain limited because of an incomplete understanding of CAF heterogeneity and identification of clinically relevant subsets. The use of single-cell RNA sequencing (scRNA-seq) has increased the resolution of the stromal cell landscape in healthy and diseased tissues. scRNA-seq analyses of CAF evolution in pancreatic ductal adenocarcinoma (PDAC) and breast cancer have identified a predominance of activated myofibroblasts (in both mice and humans) marked by LRRC15 (refs ^1–3^). This CAF population expresses a multitude of genes associated with the extracellular matrix and immunosuppression^1,9,10^. Clinically, high expression of a LRRC15^+^ CAF gene signature in bulk RNA-seq data from patients with cancer was associated with a lack of response to anti-programmed death ligand 1 (PDL1) ICB^1^. It remains unclear whether LRRC15^+^ CAFs underlie this lack of response or whether they represent a readout of tumour-intrinsic features that drive the association. Also missing is in vivo substantiation of the cellular and molecular signals that promote LRRC15^+^ myofibroblast development and their direct impact on anti-tumour immunity.

## LRRC15^+^ CAF formation depends on TGFβ receptor 2

**Fig. 1:**
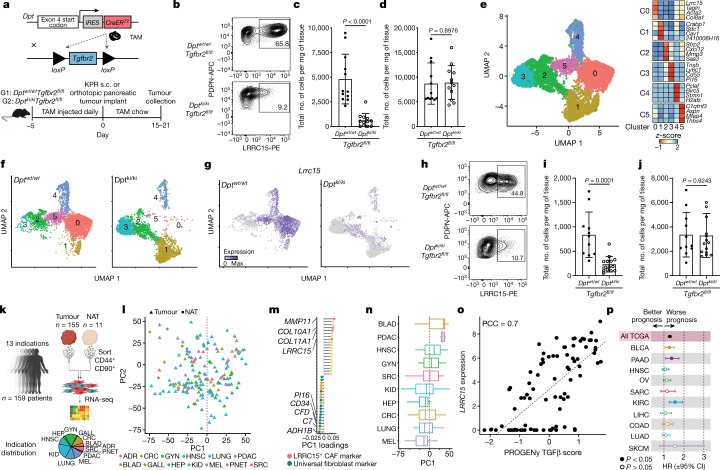
TGFβR2 signalling in DPT^+^ universal fibroblasts drives LRRC15^+^ myofibroblast differentiation. **a**, Schematic of the genetic (top) and experimental (bottom) approach for the generation of *Dpt*^*IresCreERT2*^*Tgfbr2*^*fl/fl*^ mice. s.c., subcutaneous. **b**–**g**, Data are from subcutaneous KPR tumours 21 days after implantation in *Dpt*^*wt/wt*^*Tgfbr2*^*fl/fl*^ and *Dpt*^*ki/ki*^*Tgfbr2*^*fl/fl*^ mice. **b**, Representative flow cytometry plots showing the frequency of PDPN^+^LRRC15^+^ cells. Cells were gated on PDPN^+^CD31^–^ cells. **c**,**d**, Quantification of the total number of PDPN^+^LRRC15^+^ cells (**c**) and PDPN^+^CD31^–^ cells (**d**) normalized by tumour weight (*n* = 12 mice). **e**, Uniform manifold approximation and projection (UMAP) plot of 6,525 single fibroblasts coloured by cluster membership (left, *n* = 5 mice per group) and the relative average expression of indicated marker genes in clusters (C0–C5) from the UMAP (right). **f**, UMAP as in **e** split by genotype. **g**, UMAP as in **e** split by genotype and coloured by expression of *Lrrc15*. **h**–**j**, Data are from orthotopic pancreatic KPR tumours 15 days after implantation in *Dpt*^*wt/wt*^*Tgfbr2*^fl/fl^ or *Dpt*^*ki/ki*^*Tgfbr2*^*fl/fl*^ mice **h**, Representative flow cytometry plots showing the frequency of PDPN^+^LRRC15^+^ cells. Cells were gated on PDPN^+^CD31^–^ cells. **i**,**j**, Quantification of the total number of PDPN^+^LRRC15^+^ cells (**i**) and PDPN^+^CD31^–^ cells (**j**) normalized by tumour weight (*n* = 11 or 14 mice). **k**, Scheme of collection of the human samples. NAT, normal adjacent tissue; BLAD, bladder urothelial carcinoma; GYN, gynaecologic tumours; PDAC, pancreatic ductal adenocarcinoma; HNSC, head and neck squamous cell carcinoma; SRC, sarcoma; KID, kidney cancer; HEP, liver hepatocellular carcinoma; CRC, colorectal cancer; LUNG, lung cancer; MEL, melanoma; ADR, adrenal cancer; PNET, pancreatic neuroendocrine tumour; GALL, gallbladder cancer. **l**, PCA of stromal cell samples. Shapes indicate the sample origin; colours represent the cancer indication. **m**, Gene loadings for PC1 from **l**. **n**, Distribution of samples from specified indications across PC1 from **l**. **o**, Pearson’s correlation coefficient (PCC) between *LRRC15* expression and TGFβ pathway activity across samples (filled circles). Linear regression line (dashed line). **p**, Forest plot depicting TGFβ CAF overall survival hazard ratios (HRs) across specified TCGA indications. Data in **c**, **d**, **i** and **j** are the mean ± s.d. Data in **c**, **d**, **i** and **j** are pooled from two or three independent experiments. For **n**, whiskers represent the minimum and maximum, the box represents the interquartile range, and the centre line represents the median. For **p**, the centre point shows the HR, lines represent 95% confidence interval (CI). Statistics were calculated using two-tailed, unpaired Student’s *t*-test (**c**,**d**,**i,j**) or Cox proportional hazards regression model (**p**). Source data

**Extended Data Fig. 1:**
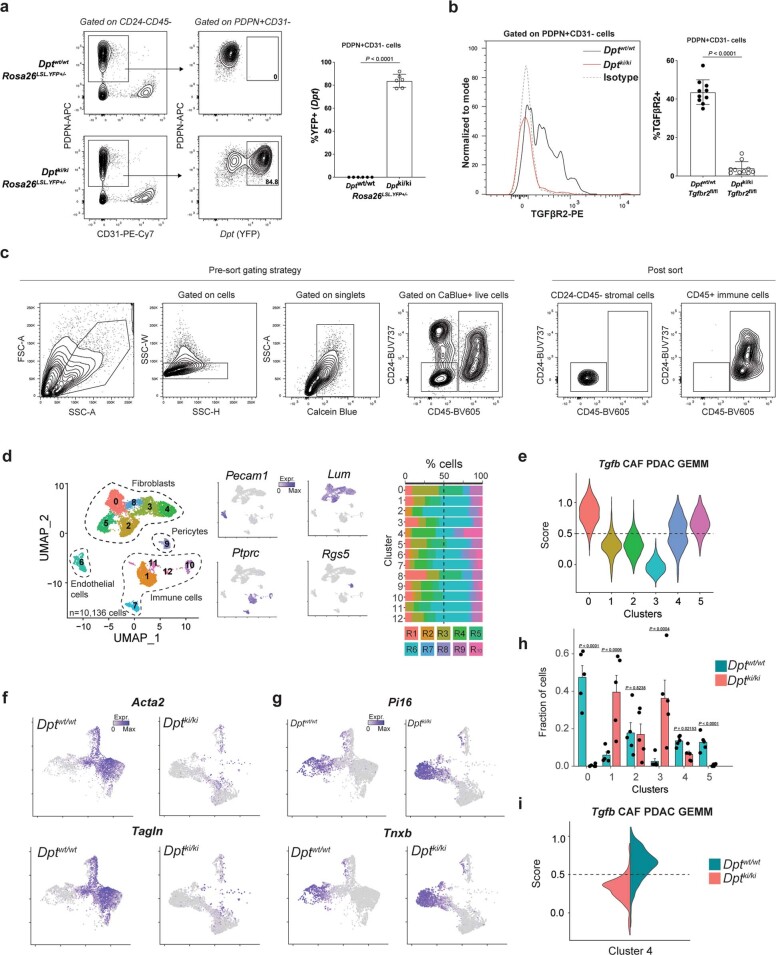
TGFβR2 staining on CAFs and scRNAseq from *Dpt*^IresCreERT2^*Tgfbr2*^fl/fl^ mice. **a**. From subcutaneous KPR tumours 21 days post implantation in *Dpt*^*wt/wt*^*Rosa26*^LSLYFP+/-^ and *Dpt*^*ki/ki*^*Rosa26*^LSLYFP+/-^ mice showing representative flow cytometry plots (left) and quantification of frequency (right) of *Dpt*-YFP^+^ cells in PDPN^+^CD31^–^ fibroblasts (n = 6 mice). **b-i**. From subcutaneous KPR tumours 21 days post implantation in *Dpt*^*wt/wt*^*Tgfbr2*^fl/fl^ and *Dpt*^*ki/ki*^*Tgfbr2*^fl/fl^ mice after experimental regimen from Fig. 1a showing **b**. Representative flow cytometry histogram (left) and quantification of frequency (right) of TGFβR2^+^ cells in PDPN^+^CD31^–^ fibroblasts (n = 10 mice). **c**. Representative pre-sort gating strategy (left) and post sort purity analysis (right) of CD24^-^CD45^-^ stromal cells and CD45^+^ immune cells **d**. UMAP plot of 10,136 stroma and immune cells colored by cluster membership (left), marker gene expression (middle), and frequency of individual cell hashed samples in clusters from UMAP (n = 5 mice per group). **e**. Violin plots showing the score for a *Tgfb* CAF PDAC GEMM gene signature for cells in each cluster from Fig. 1e. **f**,**g**. UMAPs as in Fig. 1f, colored by expression of indicated markers. **h**. Fraction of cells in each cluster from each condition from Fig. 1e (n = 5 animals per condition). **i**. Scores for the same geneset as in e, here for cells in the proliferating cluster (C4) from Fig. 1e and split by condition. Data in a,b are pooled from two or three independent experiments. Data in a,b are mean +/− s.d. and in h are mean + s.e.m. Statistics in a,b,h were calculated using a two-tailed, unpaired Student’s t-test. Source data

Recent scRNA-seq studies used in silico predictions to associate active TGFβ signalling with LRRC15^+^ myofibroblast formation during tumorigenesis^1,3^. Separate single-cell atlas studies have inferred that activated fibroblast subsets in perturbed tissues develop from pan-tissue universal fibroblasts^10^. We aimed to provide an in vivo genetic link between these two inferences, proposing that TGFβ signalling in universal fibroblasts is indispensable for the formation of LRRC15^+^ myofibroblasts during tumour progression. We used a mouse system that targets dermatopontin (DPT), an extracellular matrix protein that marks universal fibroblasts^10^. *Dpt*^*IresCreERT2*^ mice were crossed with TGFβ receptor type 2 (TGFβR2, encoded by *Tgfbr2*) floxed mice (*Dpt*^*IresCreERT2*^*Tgfbr2*^*fl/fl*^) to generate an inducible and conditional knockout of *Tgfbr2* in DPT^+^ universal fibroblasts (Fig. 1a, top). *Dpt*^*wt/wt*^*Tgfbr2*^*fl/fl*^ (control) or *Dpt*^*ki/ki*^*Tgfbr2*^*fl/fl*^ (*Dpt*-conditional knockout) mice were placed on a tamoxifen (TAM) regimen and subcutaneously implanted with KPR3070 (KPR; *Kras*^*LSL.G12D/wt*^*;p16/p19*^*fl/wt*^*;p53*^*LSL.R270H/wt*^*;Pdx1.Cre*) PDAC tumour cells^1,11^. Tumours were collected 21 days after implantation for flow cytometry analysis (Fig. 1a, bottom). To ensure efficient knockout of *Tgfbr2*, the same TAM regimen (outlined in Fig. 1a) was first performed in *Dpt*^*IresCreERT2*^*Rosa26*^*LSLYFP*^ reporter mice^10^ bearing subcutaneous KPR tumours. This was done to understand what proportion of tumour fibroblasts are derived from DPT^+^ cells, a fibroblast population abundant in naive skin tissue^10^. After 21 days of implantation, most (around 84%) of the PDPN^+^ fibroblasts in tumours were YFP^+^ (Extended Data Fig. 1a). In turn, TGFβR2 expression on PDPN^+^ CAFs from *Dpt*^*ki/ki*^*Tgfbr2*^*fl/fl*^ tumours was reduced by about 90% compared with *Dpt*^*wt/wt*^*Tgfbr2*^*fl/fl*^ tumours (Extended Data Fig. 1b). As a result, *Dpt*^*ki/ki*^*Tgfbr2*^*fl/fl*^ tumours had a significant reduction in the total number of LRRC15^+^PDPN^+^ CAFs compared with control *Dpt*^*wt/wt*^*Tgfbr2*^*fl/fl*^ tumours, which indicatedthat LRRC15^+^ cells depend on TGFβR2 signalling in DPT^+^ cells for their formation (Fig. 1b,c). Despite the significant reduction in LRRC15^+^ cells in *Dpt*^*ki/ki*^*Tgfbr2*^*fl/fl*^ tumours, the total number of PDPN^+^CD31^–^ fibroblasts was unchanged between both groups, which indicated that a compensation mechanism occurred to maintain the CAF compartment (Fig. 1d).

Maintenance of the total fibroblast content in the absence of LRRC15^+^ CAF formation in *Dpt*^*ki/ki*^*Tgfbr2*^*fl/fl*^ tumours warranted a deeper investigation of the fibroblast composition in these mice. scRNA-seq was performed on CD24^–^CD45^–^ stromal cells and CD45^+^ immune cells from both *Dpt*^*wt/wt*^*Tgfbr2*^*fl/fl*^ and *Dpt*^*ki/ki*^*Tgfbr2*^*fl/fl*^ tumours (Extended Data Fig. 1c).After quality control, dimensionality reduction and clustering, four main groups of cells were identified, each represented by cells from multiple animals (Extended Data Fig. 1d). These were immune cells (*Ptprc*^+^, also known as *Cd45*^+^), endothelial cells (*Pecam1*^+^, also known as *Cd31*^+^), pericytes (*Rgs5*^+^) and fibroblasts (*Lum*^+^) (Supplementary Table 1). Focusing our downstream analysis on fibroblasts revealed six CAF-specific clusters (Fig. 1e and Supplementary Table 2): a *Pi16*^+^ cluster that strongly expressed universal fibroblast markers (cluster 3); a *Cxcl12*^+^ cluster (cluster 2); a cluster of *Crabp1*^+^ CAFs (cluster 1); a cluster that highly expressed proliferation markers (cluster 4); and two clusters with high (cluster 0) and low (cluster 5) *Lrrc15* expression (Fig. 1e). Clusters 0 and 5 expressed additional myofibroblast markers beyond *Lrrc15*, indicative of TGFβ signalling, such as *Acta2* and *Tagln*. These two clusters also scored high for a *Tgfb* CAF gene signature previously inferred from genetically engineered mouse models (GEMMs) of PDAC^1^ (Extended Data Fig. 1e). Confirming our hypothesis that TGFβR2 signalling in DPT^+^ cells is required for LRRC15^+^ CAF development, both clusters 5 and 0 were absent in *Dpt*^*ki/ki*^*Tgfbr2*^*fl/fl*^ mice (Fig. 1f). Moreover, *Dpt*^*ki/ki*^*Tgfbr2*^*fl/fl*^ animals had almost no cells that expressed *Lrrc15*, *Acta2* or *Tagln* (Fig. 1g and Extended Data Fig. 1f). The lack of LRRC15^+^ CAFs resulted in a concomitant increase in the relative abundance of the *Pi16*^+^ universal fibroblast cluster 3 and the *Crabp1*^+^ cluster 1 in *Dpt*^*ki/ki*^*Tgfbr2*^*fl/fl*^ mice. This result corroborates our findings that the total tumour fibroblast content is compensated for in the absence of LRRC15^+^ CAF development (Extended Data Fig. 1g,h). No significant changes in the relative abundance of proliferating cluster 4 were observed, but proliferating cells from *Dpt*^*ki/ki*^*Tgfbr2*^*fl/fl*^ mice scored low for the *Tgfb* CAF PDAC GEMM signature. By contrast, cells from *Dpt*^*wt/wt*^*Tgfbr2*^*fl/fl*^ animals scored high for this signature, which is in line with our finding that LRRC15^+^ CAF formation from DPT^+^ universal fibroblasts depends on TGFβR2 (Extended Data Fig. 1i).

We next asked whether the same pathway activation is required for LRRC15^+^ CAF development in the pancreas, a tissue site with a similar abundance of DPT^+^ universal fibroblasts at steady state^10^. KPR tumour cells were orthotopically implanted into the pancreas of *Dpt*^*wt/wt*^*Tgfbr2*^*fl/fl*^ or *Dpt*^*ki/ki*^*Tgfbr2*^*fl/fl*^ mice, and tumours were analysed 15 days later (Fig. 1a, bottom). Similar to subcutaneous KPR tumours, development of LRRC15^+^PDPN^+^ CAFs in orthotopic pancreatic tumours was significantly impaired in *Dpt*^*ki/ki*^*Tgfbr2*^*fl/fl*^ mice compared with control *Dpt*^*wt/wt*^*Tgfbr2*^*fl/fl*^ mice, whereas the total number of PDPN^+^CD31^–^ fibroblasts remained unchanged (Fig. 1h–j).

These data provide direct in vivo evidence that TGFβ signalling is required for the differentiation of DPT^+^ universal fibroblasts into LRRC15^+^ myofibroblasts. Blunting LRRC15^+^ CAF development resulted in an accumulation of DPT^+^ universal fibroblasts and TGFβR2-independent CAFs to maintain the CAF compartment in their absence. Importantly, this lineage relationship and signalling dependency was required in multiple tissue sites, which confirms the universal nature of DPT^+^ fibroblasts.

## Defining a human cancer fibroblast axis

**Extended Data Fig. 2:**
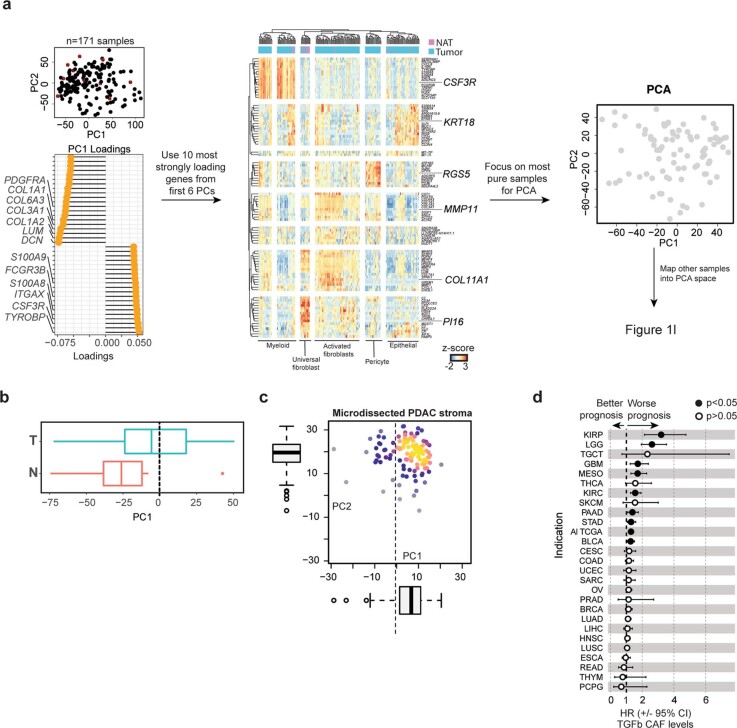
Stromal cell transcriptional analysis across human cancers. **a**. Strategy for the analysis of sorted CAF populations from human tumours: Left: PCA was performed on all samples and the 20 most strongly positive and negative loading genes from each PC were extracted. Middle: Hierarchical clustering was performed, and samples without any contamination were isolated. Right: PCA was performed on these pure samples. Samples with some contaminations were projected into this PCA space obtained from pure samples to derive Fig. 1l. **b**. Distribution of normal adjacent (N) and tumour (T) tissues along PC1 from Fig. 1l. **c**. Projection of 123 microdissected PDAC RNA-seq samples into the PCA space obtained in Fig. 1b. **d**. Same forest plot as in Fig.  1p, here for all cancer indications in TCGA. In data in b,c, whiskers represent the minimum and maximum, the box represents IQR, and the center line represents the median. In data in d, the center point shows the hazard ratio and the lines represent the 95% confidence interval. Statistics were calculated using a Cox Proportional-Hazards Regression Model in d. Source data

Next, we aimed to understand whether the axis between universal fibroblasts and LRRC15^+^ myofibroblasts was representative of the fibroblast compartment in human cancers. Previous studies^1^ relied on deconvolution of whole tumour sample bulk RNA-seq data from The Cancer Genome Atlas (TCGA) to infer abundance of a specific CAF subset across indications. To specifically focus on heterogeneity within the fibroblast compartment across human cancer types, we sorted CD45^–^CD44^+^CD90^+^ stromal cells from 159 patient samples across 13 indications (Fig. 1k and Supplementary Table 3) and generated bulk RNA-seq expression profiles. Filtering for high-purity samples on the basis of principal component analysis (PCA) and hierarchical clustering enabled an unbiased view into human CAF expression programmes and their pan-cancer prevalence (Extended Data Fig. 2a). We did not observe a clear separation of cancer indications in the PCA space, which suggested that pan-cancer stromal cell signatures were driving the two first principal components (PCs) (Fig. 1l). Genes with the strongest positive weights for PC1 comprised known LRRC15^+^ CAF expression marker genes such as *COL10A1*, *COL11A1*, *MMP11* and *LRRC15* (ref. ^1^). Conversely, markers associated with previously described universal fibroblasts, such as *CD34 and* *PI16*, had the strongest negative weights for PC1 (ref. ^10^) (Fig. 1m). Consistently, samples obtained from normal adjacent tissue exhibited mostly negative PC1 values (Extended Data Fig. 2b). Among the indications analysed, pancreatic cancer and bladder cancer samples had the highest PC1 values, which indicated strong enrichment of LRRC15^+^ CAFs (Fig. 1n). The trend of high LRRC15^+^ CAF levels in pancreatic cancer was confirmed in an independent dataset (Extended Data Fig. 2c). Pathway enrichment analysis revealed that samples with high levels of *LRRC15* expression showed increased TGFβ pathway activation, thereby strongly suggesting that the LRRC15^+^ CAF expression programme in humans, as genetically demonstrated in our mouse model, is similarly driven by TGFβ signalling (Fig. 1o). High expression levels of TGFβ CAF markers were significantly associated with worse survival across samples from all indications in TCGA and within certain tumour types, including bladder cancer and pancreatic cancer (Fig. 1p and Extended Data Fig. 2d). These human data reveal a central axis of CAF heterogeneity across human cancers defined by universal fibroblasts and LRRC15^+^ myofibroblasts, with TGFβ signalling enriched in indications in which LRRC15^+^ CAFs are abundant.

Together with the results from our genetic mouse system, these findings suggest that TGFβ signalling acts a rheostat to dictate the tumour fibroblast setpoint between universal fibroblasts and LRRC15^+^ myofibroblasts and may serve as a potential predictor of patient outcome.

## LRRC15 expression is restricted to CAFs

**Fig. 2:**
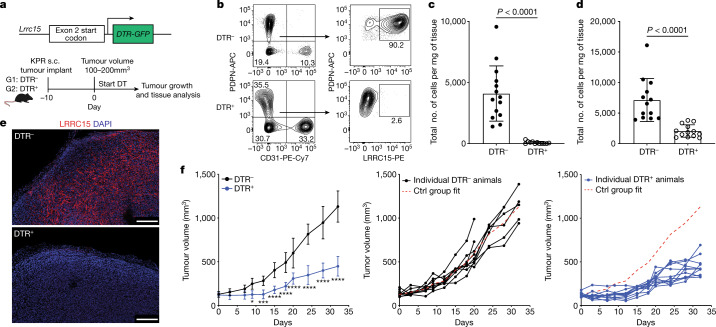
Targeted depletion of LRRC15^+^ CAFs significantly reduces tumour growth. **a**, Schematic of the genetic (top) and experimental (bottom) approach for *Lrrc15*^*DTRGFP*^ mice. **b**–**e**, Data are from subcutaneous KPR tumours 8 days after DT treatment in DTR^–^ and DTR^+^ mice. **b**, Representative flow cytometry plots showing the frequency of PDPN^+^LRRC15^+^ cells. Cells were gated on CD24^–^CD45^–^ cells (left) or PDPN^+^CD31^–^ cells (right). **c**,**d**, Quantification of the total number of PDPN^+^LRRC15^+^ cells (**c**) and PDPN^+^CD31^–^ cells (**d**) normalized by tumour weight (*n* = 12 or 14 mice). **e**, Representative immunofluorescence images of LRRC15 and DAPI. Scale bar, 250 µm. **f**, Tumour growth curves from DTR^–^ and DTR^+^ mice treated with DT (*n* = 9 or 11 mice per group). Left: average tumour volume across all animals (*P = 0.015, ***P = 0.0006, ****P < 0.0001). Middle and right: individual animal growth curves per genotype. The *x* axis represents days after DT treatment. The dashed red line represents the average reference fit for the control (Ctrl) group. Data in **c** and **d** are pooled from four independent experiments. Data in **e** and **f** are representative of two or three independent experiments. Data in **c**, **d** and **f** are the mean ± s.d. Statistics were calculated using two-tailed, unpaired Student’s *t*-test (**c** and **d**) or ordinary two-way analysis of variance (**f**). Source data

**Extended Data Fig. 3:**
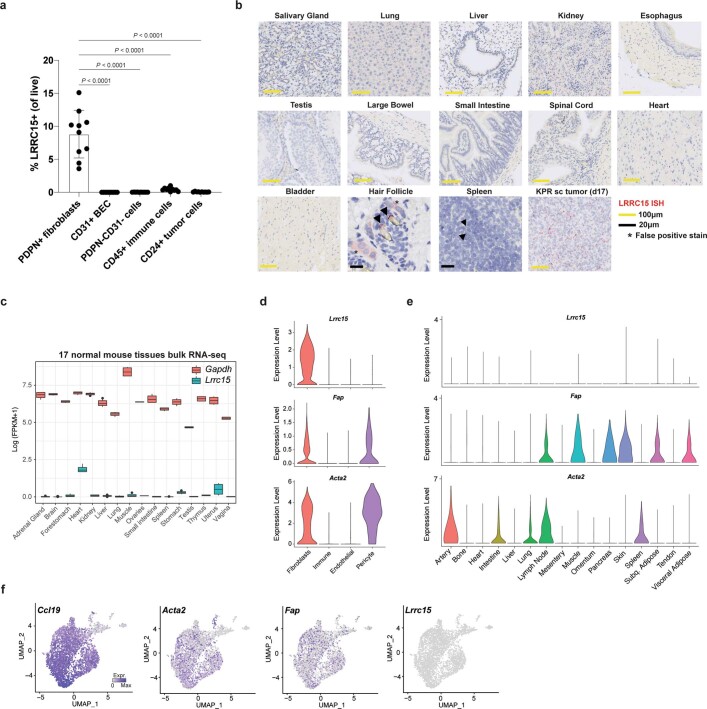
LRRC15 expression is restricted to tumour-associated fibroblasts. **a**. Quantification of frequency of LRRC15^+^ cells in subcutaneous KPR tumours 17 days post implantation in PDPN^+^ fibroblasts, CD31^+^ blood endothelial cells (BEC), PDPN^–^CD31^–^ stromal cells, CD45^+^ immune cells, and CD24^+^ tumour cells (n = 10 mice). **b**. Representative LRRC15 *in situ* hybridization images of select healthy murine tissues and subcutaneous KPR tumour tissue 17 days post implantation. **c**. Bulk RNAseq data of 17 normal mouse tissues showing *Lrrc15 and Gapdh* (control) expression. Number of samples per tissue are in the source data. **d-f**. *Fap, Acta2, and Lrrc15* gene expression from **d**. scRNAseq analysis of fibroblasts, immune cells, endothelial cells, and pericytes from KPR tumours 21 days post implantation in *Dpt*^*wt/wt*^*Tgfbr2*^fl/fl^ mice. **e**. scRNAseq of *Pdgfrα*^*+*^ steady state fibroblasts across murine tissues. **f**. scRNAseq of fibroblasts and pericytes from murine naive skin-draining lymph nodes along with *Ccl19* expression. Data in a are pooled from three independent experiments. Data in b are representative of one independent experiment. Data in a are mean +/− s.d. In data in c, whiskers represent the minimum and maximum, the box represents IQR, and the center line represents the median. Statistics in a were calculated using an ordinary one-way ANOVA test. Source data

To investigate the impact of LRRC15^+^ CAFs on tumour growth and anti-tumour immunity, a genetic mouse model was generated wherein a diphtheria toxin receptor (DTR)–GFP cassette was knocked into exon 2, downstream of the start codon of *Lrrc15* (*Lrrc15*^*DTRGFP*^ knock-in mice). This model enabled the controlled depletion of LRRC15-expressing cells following the administration of diphtheria toxin (DT) (Fig. 2a, top). To ensure that this approach would provide selective ablation of this subpopulation of CAFs, we evaluated LRRC15 expression in mice. Within KPR tumours, LRRC15 expression was restricted to PDPN^+^ fibroblasts and largely absent in other compartments (Extended Data Fig. 3a). Outside tumours, in situ hybridization and bulk RNA-seq^12^ analysis showed that *Lrrc15* expression was low to absent across multiple tissues (Extended Data Fig. 3b,c). Similar results have been reported in human tumours and peripheral tissues^13^.

As previous CAF-depletion strategies have used markers such as α-smooth muscle actin (αSMA, encoded by *Acta2*) and fibroblast activation protein (FAP, encoded by *Fap*)^14,15^, we compared expression levels of *Fap* and *Acta2* to *Lrrc15*. In KPR tumours, *Lrrc15* expression was restricted to fibroblasts, whereas *Acta2* and *Fap* expression was observed in both fibroblasts and pericytes (Extended Data Fig. 3d). Beyond the tumour, expression of both *Fap* and *Acta2* was observed in stromal cells across multiple tissues, whereas *Lrrc15* was absent (Extended Data Fig. 3e). In mouse skin-draining lymph nodes (LNs), *Acta2* was highly expressed by pericytes, and both *Fap and Acta2* expression prominently overlapped with *Ccl19*^+^ fibroblastic reticular cells. By contrast, *Lrrc15* was undetectable in LN fibroblastic reticular cells or pericytes^16^ (Extended Data Fig. 3f). Collectively, these data demonstrate that LRRC15 is a bona fide marker of TGFβ-driven CAFs that does not overlap with other cells within and beyond tumours.

## LRRC15^+^ CAF depletion slows tumour growth

**Extended Data Fig. 4:**
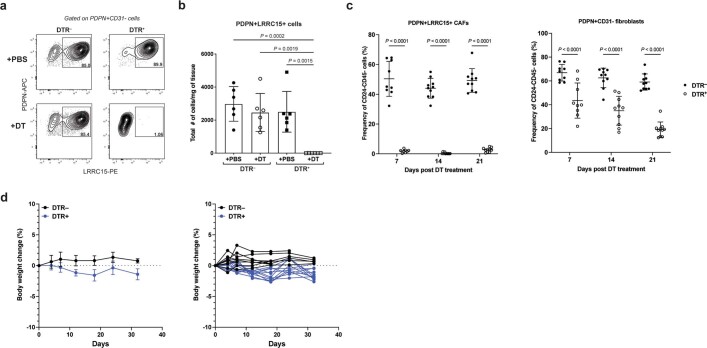
Selective depletion of LRRC15^+^ CAFs is achieved in *Lrrc15*^DTR^ mice. **a,b**. From subcutaneous KPR tumours 8 days post PBS or DT treatment in DTR^–^ and DTR^+^ mice. **a**. Representative flow cytometry plots showing frequency of LRRC15^+^ cells in PDPN^+^CD31^–^ fibroblasts and **b**. Quantification of total number of PDPN^+^LRRC15^+^ cells normalized by tumour weight (n = 6 mice) **c**. Quantification of frequency of PDPN^+^LRRC15^+^ cells (left) and PDPN^+^CD31^–^ fibroblasts (right) on days 7 (n = 8 or 9 mice), 14 (n = 10 mice), and 21 (n = 10 mice) post DT treatment in subcutaneous KPR tumour bearing DTR^–^ and DTR^+^ mice. **d**. Body weight change curves from DTR^–^ and DTR^+^ mice treated with DT (n = 9 or 11 mice/group). Average body weight change across all animals (left); Individual body weight change curves per genotype (right). X-axis represents days post DT treatment. Data in b,c are pooled from two or three independent experiments. Data in d are representative of three independent experiments. Data in b,c,d are mean +/− s.d. Statistics were calculated using an Ordinary one-way ANOVA test in b and an Ordinary two-way ANOVA in c. Source data

Given the specificity of *Lrrc15* expression, we proceeded to assess the impact of selectively depleting LRRC15^+^ CAFs on tumour growth. KPR tumours were subcutaneously implanted into *Lrrc15*^*DTRGFPwt/wt*^ (DTR^–^) or *Lrrc15*^*DTRGFPwt/ki*^ (DTR^+^) mice, and DT treatment was initiated in both groups of mice when tumours reached a mean volume of 100–200 mm^3^ (around 10 days after implantation) (Fig. 2a, bottom). Eight days later, tumours were collected and evaluated for the presence of LRRC15^+^ CAFs. Tumours from DT-treated DTR^–^ mice had a predominant population of LRRC15^+^ CAFs, whereas DT treatment in DTR^+^ tumours resulted in an approximately 98% loss of total LRRC15^+^ cells (Fig. 2b,c). Importantly, loss of LRRC15^+^ CAFs in DTR^+^ mice was specific to DT treatment and not a result of insufficient development of these cells in the absence of DT (Extended Data Fig. 4a,b). Total PDPN^+^ fibroblast numbers were also significantly reduced by about 70% in DT-treated DTR^+^ mice (Fig. 2d). Immunofluorescence imaging confirmed these results, showing an absence of LRRC15 staining in DTR^+^ tumours (Fig. 2e). Continued DT administration sustained significant LRRC15^+^ CAF depletion and a diminished PDPN^+^ fibroblast compartment beyond day 8 and did not cause any significant body weight changes in either group of mice (Extended Data Fig. 4c,d). As a result, tumour growth was significantly slowed following sustained depletion of LRRC15^+^ CAFs in DTR^+^ mice compared with DTR^–^ control mice (Fig. 2f). Collectively, these data show that selective ablation of the LRRC15^+^ subtype of CAFs in tumours leads to a significant decrease in total CAF content and a marked and persistent reduction in tumour burden.

## Recalibrating the tumour fibroblast setpoint

**Fig. 3:**
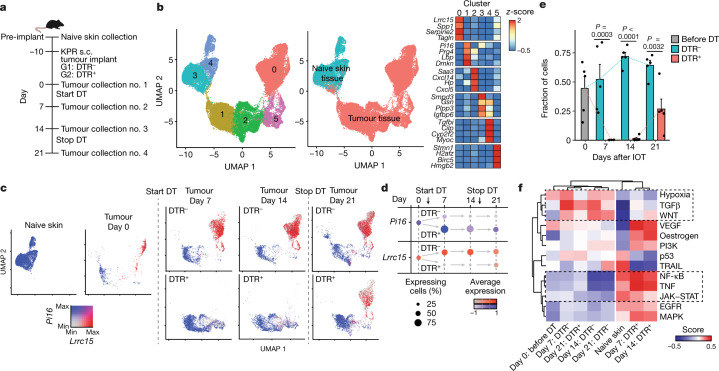
Normal tissue universal fibroblast-like activity is enriched following LRRC15^+^ CAF depletion. **a**, Experimental scheme (*n* = 5 mice per timepoint and group). **b**, UMAP plot of 37,383 single fibroblasts coloured by cluster membership (left) or coloured by tissue of origin (middle). Relative average expression of indicated marker genes across clusters from left UMAP (right). **c**, UMAP as in **b**, coloured by expression of *Pi16* and *Lrrc15* and split by time point and condition. **d**, Dot plot visualizing the percentage of positive fibroblasts (dot size) and the relative average expression (colour) for *Lrrc15* and *Pi16* at each time point and condition in tumour-bearing samples. **e**, Fraction of cells in cluster 0 of each treatment group (*n* = 5 mice per group) at all four time points in tumour-bearing samples. **f**, PROGENy pathway enrichment scores (colour) for cells pooled for each of the indicated time points and conditions (bottom row). Data in **e** are the mean + s.e.m., and statistics were calculated using two-tailed, unpaired Student’s *t*-test. Source data

**Extended Data Fig. 5:**
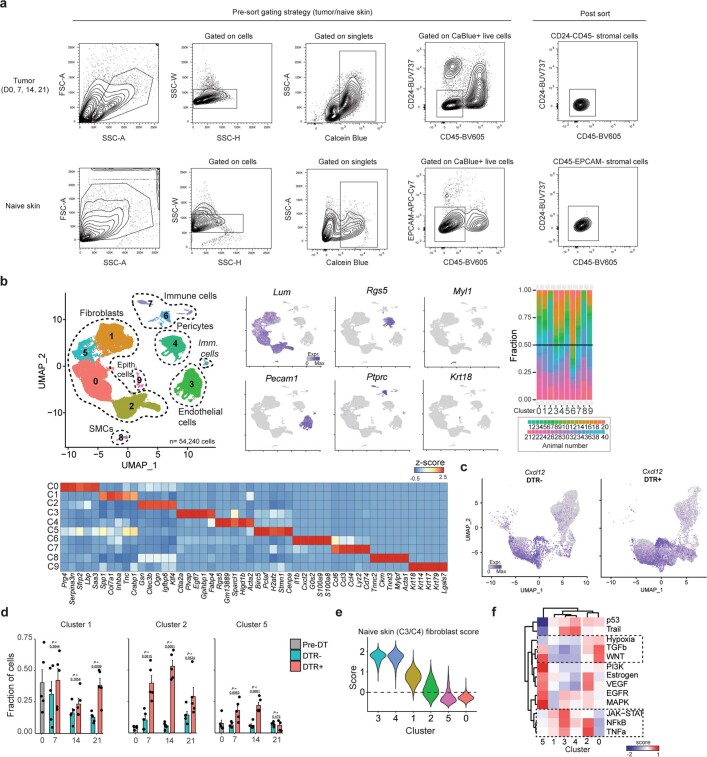
Kinetic scRNAseq of naïve skin fibroblasts and tumour-associated fibroblasts following LRRC15^+^ CAF ablation. **a**. Representative pre-sort gating strategy (left) and post sort purity analysis (right) of stromal cells from subcutaneous KPR tumour tissue (top) or naive skin tissue (bottom). **b**. UMAP plot of 54,240 stromal cells colored by cluster membership (left). UMAP as on the left, here colored by indicated marker gene expression (middle) and frequency of individual cell hashed samples in clusters from UMAP (right). Relative average expression of top 5 marker genes in each cluster (bottom). **c**. UMAP (D0,7,14,21; left, D7,14,21; right) as in 3b split colored by expression of *Cxcl12*. **d**. Fraction of cells in C1, C2, and C5 from Fig. 3b in each condition (n = 5 animals/condition) at all four time points in tumour bearing samples. **e**. Violin plots showing the score for a naive skin C3/C4 gene signature for cells from each cluster in Fig. 3b. **f**. PROGENY pathway enrichment scores (color) for cells pooled for each cluster in Fig. 3b. Data in d are mean + s.e.m. Statistics in d were calculated using a two-tailed, unpaired Student’s t-test. Source data

The significant impact of LRRC15^+^ CAF ablation on the fibroblast compartment led us to investigate the composition of the remaining CAF environment in their absence. scRNA-seq of CD24^–^CD45^–^ stromal cells from tumours in DTR^–^ and DTR^+^ mice was carried out at 4 different time points, including 10 days after tumour implantation and immediately before initiation of DT treatment (IOT) (day 0) and on days 7, 14 and 21 after IOT (Fig. 3a and Extended Data Fig. 5a, top). DT treatment was initiated in all mice from day 0 to day 14 and then stopped for the last week of the study to day 21 (Fig. 3a). Additionally, EPCAM^–^CD45^–^ stromal cells from naive, non-tumour-bearing skin tissue were characterized to establish a baseline profile before tumour implantation (Fig. 3a and Extended Data Fig. 5a, bottom).

After quality control, 54,240 single stromal cells were analysed across all time points and treatment groups. Dimensionality reduction and clustering revealed pericytes (*Rgs5*^+^), endothelial cells (*Pecam1*^+^, also known as *Cd31*^+^) and fibroblasts (*Lum*^+^) as the three main stromal cell populations (Extended Data Fig. 5b, left and bottom, and Supplementary Table 4). All clusters were populated by cells from multiple animals, and subsequent analyses focused on fibroblasts (Extended Data Fig. 5b, right, and Supplementary Table 5). Fibroblasts from naive skin tissue formed two separate clusters (clusters 3 and 4) with little to no admixture of cells from tumour-bearing mice (Fig. 3b). Within tumour-bearing tissue, fibroblasts could be assigned to four transcriptional expression phenotypes: an *Lrrc15* cluster (cluster 0) that also expressed myofibroblast markers such as *Tagln* and *Spp1*; a cluster of proliferating CAFs (cluster 5); a cluster of *Cxcl14*-expressing CAFs (cluster 2); and a cluster of *Pi16*^high^ CAFs (cluster 1) that shared expression patterns with universal fibroblasts^10^ (Fig. 3b).

Fibroblast dynamics in DT-treated DTR^–^ and DTR^+^ mice were then monitored, and the expression of *Pi16* and *Lrrc15* at each time point was compared (Fig. 3c). In naive, non-tumour-bearing skin, *Lrrc15* expression was absent and all cells were uniformly *Pi16*^+^. This result indicated a universal fibroblast phenotype that was similar to that of normal pancreatic tissue fibroblasts^1,10^. In tumour tissue, on day 0, *Pi16*^+^ cells and *Lrrc15*^*+*^ cells were detected. In DTR^–^ animals, LRRC15^+^ CAFs emerged as the dominant CAF population throughout the time course, starting at day 7 and persisting up to day 21 (Fig. 3c,d). Conversely, in DTR^+^ animals, *Lrrc15*^*+*^ cells were absent during the first 2 weeks of DT treatment, and a concomitant relative increase in *Pi16*^+^ cells, of which a subset also expressed *Cxcl12*, was observed (Fig. 3c,d and Extended Data Fig. 5c). On day 21, 1 week after cessation of DT treatment, *Lrrc15*^+^ cells re-emerged (Fig. 3c,d). This same pattern of *Lrrc15* kinetics was reflected on the cluster level, in which the frequency of cells from LRRC15^+^ CAF cluster 0 increased and was maintained in DTR^–^ animals. By contrast, DTR^+^ animals were depleted of cluster 0 CAFs before re-emergence following DT removal (Fig. 3e). The relative frequencies of tumour-associated clusters 1, 2 and 5 were also increased in DTR^+^ animals (Extended Data Fig. 5d). Partially retained *Pi16* expression by cluster 1, 2 and 5 CAFs suggested that they are in a state that is more similar to normal tissue fibroblasts. In support of this observation, clusters 1 and 2 scored higher for a signature of cluster 3 and 4 normal skin fibroblasts than for clusters 0 and 5 (Extended Data Fig. 5e).

PROGENy pathway activity analysis^17^ revealed high TGFβ activity in samples in which LRRC15^+^ CAFs were present. By contrast, samples in which LRRC15^+^ CAFs were depleted were most similar to fibroblast samples from naive skin and enriched for JAK–STAT, NF-κB and TNF signalling pathways (Fig. 3f). This was largely explained by the abovementioned changes in cluster abundance, in which clusters 1 and 2 shared JAK–STAT, NF-κB and TNF signalling pathways with normal tissue fibroblasts (clusters 3 and 4) compared with cluster 0, which had highest TGFβ activity (Extended Data Fig. 5f). Together, these data indicate that depletion of LRRC15^+^ CAFs not only reduces overall fibroblast content in KPR tumours but also recalibrates the setpoint of the remaining CAFs towards a more universal fibroblast-like state.

## LRRC15^+^ CAFs impede CD8^+^ T cell function

**Fig. 4:**
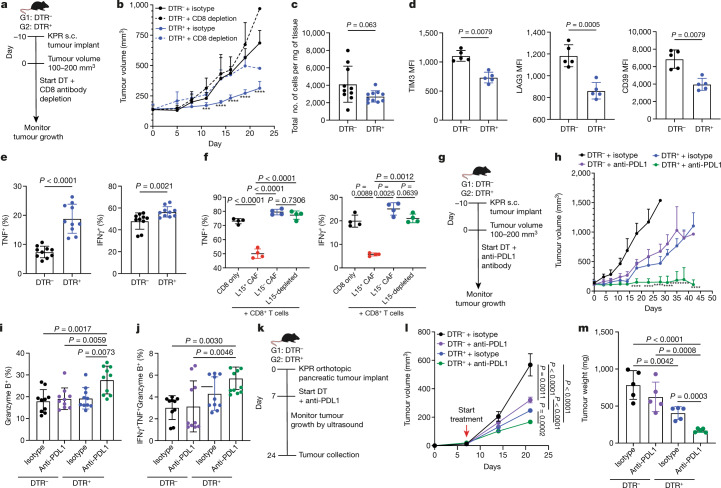
LRRC15^+^ CAF depletion enhances CD8^+^ T cell effector function and responsiveness to anti-PDL1 treatment. **a**,**b**, Data are from DTR^–^ and DTR^+^ mice bearing subcutaneous KPR tumours treated with DT and a CD8-depleting antibody. **a**, Experimental scheme. **b**, Average tumour growth curves (*n* = 10 mice per group; *** *P* = 0.0002, **** *P* < 0.0001). **c**–**e**, Subcutaneous KPR tumour analysis on day 12 after DT treatment in DTR^–^ and DTR^+^ mice. **c**, Quantification of CD8^+^ T cells normalized by tumour weight (*n* = 10 mice). **d**, Quantification of mean fluorescence intensity (MFI) of TIM3, LAG3 and CD39 on CD8^+^PD1^+^ T cells (*n* = 5 mice). **e**, Quantification of the frequency of TNF^+^ and IFNγ^+^ CD8^+^ T cells (*n* = 10 mice). **f**, Quantification of the frequency of TNF^+^ and IFNγ^+^ of anti-CD3 and anti-CD28-activated CD8^+^ T cells after 72 h of culture alone or with sorted CAFs (L15, LRRC15; *n* = 4 samples). **g**–**j**, Data are from DTR^–^ and DTR^+^ mice bearing subcutaneous KPR tumours treated with DT and an anti-PDL1 antibody. **g**, Experimental scheme. **h**, Average tumour growth curves (*n* = 9 or 10 mice per group; ****P < 0.0001). **i**,**j**, Subcutaneous KPR tumour analysis 12 days after treatment showing quantification of frequency of granzyme B^+^ CD8^+^ T cells (*n* = 10 mice) (**i**) and TNF^+^IFNγ^+^granzyme B^+^ CD8^+^ T cells (*n* = 10 mice) (**j**). **k**–**m**, Data are from DTR^–^ and DTR^+^ mice bearing orthotopic pancreatic KPR tumours treated with DT and an anti-PDL1 antibody **k**, Experimental scheme. **l**, Average tumour growth curves (*n* = 7 mice per group). The *x* axis represents days after implantation. **m**, Tumour weight on day 24 after implantation (*n* = 5 mice). Data in **b**–**f**, **h**–**j**, **l** and **m** are the mean ± s.d. Data in **b**, **h**, **d** and **f** are representative of two independent experiments and **l** and **m** are representative of one independent experiment. Data in **c**, **e**, **I** and **j** are pooled from two independent experiments. Statistics were calculated using ordinary one-way analysis of variance test (**b**,**f**,**h**–**j**,**l,m**) or two-tailed, unpaired Student’s *t*-test (**c**,**d**,**e**). Significance values mark the DTR^+^ + isotype group in **b** and the DTR^+^ + anti-PDL1 group in **h** relative to the other three groups. Source data

**Extended Data Fig. 6:**
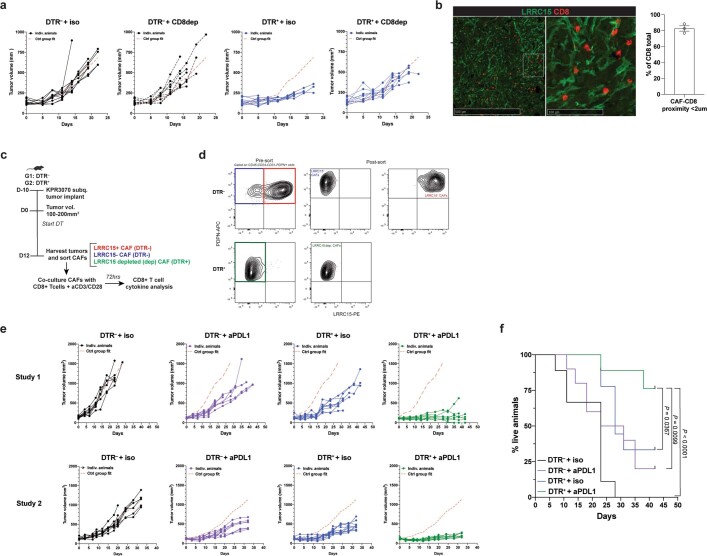
Tumour growth curves in LRRC15^+^ CAF depleted tumours combined with CD8^+^ T cell depletion or anti-PDL1 treatment and CAF sorting strategy. **a**. Individual tumour growth curves from DT-treated subcutaneous KPR tumour bearing DTR^–^ and DTR^+^ mice in combination with CD8 depleting antibody or isotype control (n = 10 mice/group). Dashed red line represents the average reference fit from the control group (DTR^–^ + isotype). **b**. Immunofluorescence analysis of subcutaneous KPR tumours stained for LRRC15 and CD8 (left) and quantification of frequency of CD8^+^ T cells in a proximity of less than 2um from an LRRC15^+^ CAF. **c,d**. Experimental scheme (c) and sorting strategy (d) for CAF-CD8^+^ T cell co-culture experiment for results in 4f. **e,f**. From DT-treated subcutaneous KPR tumour bearing DTR^–^ and DTR^+^ mice in combination with aPDL1 antibody or isotype control **e**. Two independent studies showing individual tumour growth curves (Study 1: n = 9 or 10 mice/group; Study 2: n = 9 or 11 mice/group). Dashed red line represents the average reference fit from the control group (DTR^–^ + isotype). **f**. Survival analysis of time to progression to tumour volumes reaching 1000m^3^ or tumour ulceration bigger than 5mm (n = 9 or 10 mice/group). Data in b are mean +/− s.e.m. Data in a,f are representative of two independent experiments. Statistics in f were calculated using a Log-rank Mantel-Cox test. Source data

Recent studies have identified a clinical association between high expression of a LRRC15^+^ CAF signature and lack of response to anti-PDL1 treatment across multiple cancer types^1,2^. However, it remains untested whether LRRC15^+^ CAFs are the direct cause of this association. We proposed that T cell immunity and ICB responsiveness would be affected in the absence of LRRC15^+^ CAFs and tested this in our preclinical model. First, we determined whether the improved tumour control observed following LRRC15^+^ CAF ablation depends on CD8^+^ T cells. To this end, DTR^–^ and DTR^+^ mice bearing subcutaneous KPR tumours and treated with DT were also given a CD8-depleting or isotype control antibody. Tumour growth was monitored over the course of treatment (Fig. 4a). Mice in which LRRC15^+^ CAFs were depleted exhibited significantly reduced tumour burden compared with mice with sufficient LRRC15^+^ CAFs (Fig. 4b and Extended Data Fig. 6a). Depletion of CD8 T cells reversed this effect, which indicated that CD8^+^ T cells have a role in reducing tumour burden in the absence of LRRC15^+^ CAFs (Fig. 4b and Extended Data Fig. 6a).

To understand the pharmacodynamic effects of LRRC15^+^ CAF depletion on T cell function, we used flow cytometry to characterize the intratumoural CD8^+^ T cell compartment 12 days after CAF depletion. No difference in the total number of intratumoural CD8^+^ T cells between LRRC15^+^ CAF-sufficient and CAF-deficient tumours was observed (Fig. 4c). However, in the absence of LRRC15^+^ CAFs, PD1^+^CD8^+^ T cells exhibited significantly reduced surface marker expression of molecules associated with T cell exhaustion and dysfunction^18,19^, including TIM3, LAG3 and CD39 (Fig. 4d). Moreover, we observed an enhancement in CD8^+^ T cell function, as shown by the increased expression of TNF and IFNγ (Fig. 4e).

Immunofluorescence analysis of KPR tumours revealed a significant proportion of tumour-infiltrating CD8^+^ T cells in close proximity with LRRC15^+^ CAFs, which suggested that direct cell-to-cell interactions were occurring (Extended Data Fig. 6b). This led us to ask whether LRRC15^+^ CAFs can directly influence effector T cell potential. LRRC15^+^ and LRRC15^–^ CAFs from DTR^–^ tumours or PDPN^+^ LRRC15-depleted CAFs from DTR^+^ tumours were sorted 12 days after DT treatment. These were then individually co-cultured with splenic CD8^+^ T cells in the presence of anti-CD3 and anti-CD28 antibodies (Extended Data Fig. 6c,d). Three days later, CD8^+^ T cells were re-stimulated and assessed for TNF and IFNγ protein expression. Compared with CD8^+^ T cells alone, TNF and IFNγ expression was significantly reduced in the presence of LRRC15^+^ CAFs, whereas T cell function was unchanged in the presence of LRRC15^–^ CAFs or the normalized LRRC15-depleted CAFs (Fig. 4f). These results demonstrate a role for LRRC15^+^ CAFs in repressing intratumoural CD8^+^ T cell function and show that LRRC15^+^ CAFs can directly limit CD8^+^ T cell effector potential.

## LRRC15^+^ CAF ablation boosts ICB responses

Previous work has shown that depletion of FAP^+^ myofibroblasts in cancer models can improve anti-PDL1 responsiveness^20^. We wanted to test whether similar effects are observed following LRRC15^+^ CAF ablation. DTR^–^ and DTR^+^ mice bearing subcutaneous KPR tumours and treated with DT were given an anti-PDL1 or an isotype control antibody, and tumour growth was evaluated (Fig. 4g). LRRC15^+^ CAF-sufficient mice showed some sensitivity to anti-PDL1 treatment, as demonstrated by a partial reduction in tumour burden. Conversely, responsiveness to anti-PDL1 treatment was significantly potentiated in LRRC15^+^ CAF-depleted mice, as reflected in the more substantial reduction in tumour burden. (Fig. 4h and Extended Data Fig. 6e). This combination setting not only improved tumour control but also led to a significant survival benefit, as measured by the time to progression of tumours (Extended Data Fig. 6f). Flow cytometry analysis of the CD8^+^ T cell compartment 12 days after anti-PDL1 treatment with LRRC15^+^ CAF depletion revealed an increase in their cytolytic potential, as measured by granzyme B expression. The frequency of polyfunctional T cells was also increased, as reflected by the number of TNF^+^IFNγ^+^granzyme B^+^ CD8^+^ T cells (Fig. 4i,j).

**Extended Data Fig. 7:**
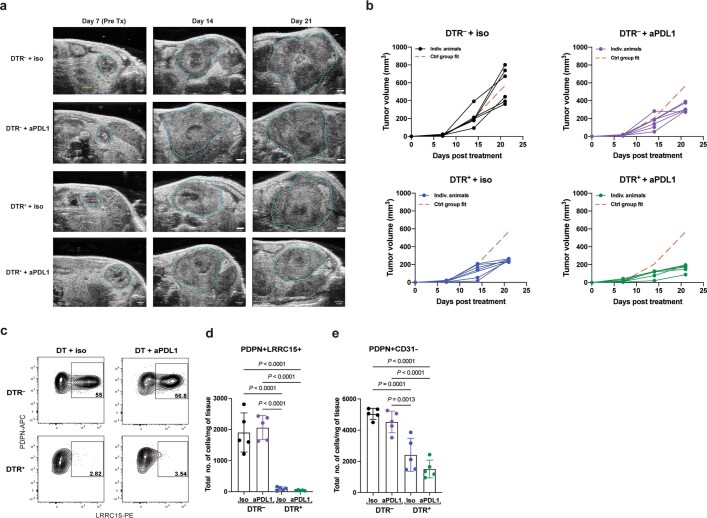
Tumour growth curves and CAF analysis in LRRC15^+^ CAF depleted orthotopic pancreatic KPR tumours combined with anti-PDL1 treatment. **a–e**. From DT-treated orthotopic pancreatic KPR tumour bearing DTR^–^ and DTR^+^ mice in combination with aPDL1 antibody or isotype control **a**. Representative ultrasound images of pancreatic KPR tumours across all treatment groups and timepoints. Hashed blue line marks circumference of tumour. **b**. Individual tumour growth curves (n = 7 mice/group). Dashed red line represents the average reference fit from the control group (DTR^–^ + isotype). **c**. Representative flow cytometry plots showing frequency of LRRC15^+^ cells in PDPN^+^CD31^–^ fibroblasts **d,e**. Quantification of total number of PDPN^+^LRRC15^+^ cells (d) and PDPN^+^CD31^+^ cells (e) normalized by tumour weight (n = 5 mice). Data in d,e are mean +/− s.d. Data in a-e are representative of one independent experiment. Statistics in d,e were calculated using an ordinary one-way ANOVA test. Source data

We next asked whether the absence of LRRC15^+^ CAFs improved the responsiveness to anti-PDL1 treatment in KPR tumours grown in the pancreas. KPR tumour cells were orthotopically implanted into the pancreas of DTR^–^ or DTR^+^ mice. On day 7 after implantation, DT treatment in combination with anti-PDL1 antibody or an isotype control was initiated, and tumour burden was measured by ultrasound imaging (Fig. 4k and Extended data Fig. 7a). Similar to the subcutaneous tumours, ablation of LRRC15^+^ CAFs in orthotopic pancreatic tumours significantly improved tumour control and synergized with anti-PDL1 to further reduce tumour burden (Fig. 4l and Extended data Fig. 7b). Tumours collected on day 24 from DTR^+^ mice treated with anti-PDL1 showed significantly lower tumour weights than control mice, which reflected the tumour volume kinetics observed during treatment (Fig. 4m). Furthermore, DTR^+^ pancreatic tumours, whether treated with anti-PDL1 or isotype control, showed a near complete ablation of LRRC15^+^ CAFs and a significant reduction in the total PDPN^+^ fibroblast compartment (Extended Data Fig. 7c-e). Together, these findings show that therapeutic depletion of LRRC15^+^ CAFs from the pancreatic tumour microenvironment leads to markedly improved responsiveness to anti-PDL1 ICB treatment.

## Discussion

In this study, we provided direct genetic evidence that TGFβ signalling in DPT^+^ universal fibroblasts promotes LRRC15^+^ myofibroblast formation during tumorigenesis, constituting a central fibroblast axis in multiple human cancers. Selective depletion of LRRC15^+^ CAFs markedly reduced the total fibroblast content and reverted this stromal compartment to a universal fibroblast-like state. In turn, this enhanced intratumoural CD8^+^ T cell effector function and potentiated anti-PDL1 responsiveness.

The utility of LRRC15 as a highly restricted marker for this myofibroblast subset enabled us to directly investigate their role in shaping the TME without perturbing fibroblasts in other tissues, such as LNs, where tissue architecture and T cell priming and function are shaped by the local fibroblastic network^16,21^. Analyses of LRRC15^+^ CAF-depleted tumours revealed that universal fibroblast-like activity was enriched but without sustained ablation, LRRC15^+^ myofibroblasts can replenish and re-establish their foothold in the CAF compartment. These data highlight the ‘push and pull’ relationship that LRRC15^+^ CAFs have with universal fibroblasts to establish a tumour fibroblast setpoint that ultimately suppresses anti-tumour T cell immunity and the effectiveness of ICB therapy. Immunologically, further investigation is warranted to understand the nature of the relationship between CD8^+^ T cells and LRRC15^+^ CAFs that leads to their functional suppression. Additionally, it will be important to understand whether LRRC15^+^ CAFs dictate ICB responsiveness similarly across different indications and tumour immune phenotypes.

Therapeutically, our findings raise the issue of the optimal strategy to modulate LRRC15^+^ CAF activity. The use of TGFβ inhibitors, which are currently being evaluated in multiple clinical trials^22^, is an attractive therapeutic option to impair LRRC15^+^ CAF formation. However, the removal of TGFβ signalling in DPT^+^ precursors enabled a compensatory mechanism to maintain total fibroblast numbers. Conversely, LRRC15^+^ CAF ablation markedly reduced fibroblast cellularity in the TME. If the optimal environment for an effective anti-tumour immune response requires a CAF compartment that is both smaller and devoid of LRRC15^+^ CAFs, our data strongly suggest that depleting LRRC15^+^ CAFs themselves may be a more attractive therapeutic strategy to generate robust and durable responses to cancer immunotherapy. Moreover, the presence of LRRC15^+^ myofibroblasts in other, non-neoplastic diseases, such as idiopathic pulmonary fibrosis and ulcerative colitis^10^, suggests that such a therapeutic approach may be broadened to provide patient benefit in other disease areas.

## Methods

### Mice

*Dpt*^*IresCreERT2*^ mice^10^ and *Lrrc15*^*DTRGFP*^ mice were designed, generated and bred at Genentech. *Tgfbr2*^*fl/fl*^ mice (012603) were obtained from the Jackson Laboratory. Age- and sex-matched mice (6–12 weeks old) were used for all studies. Mice were maintained under specific pathogen-free conditions using the guidelines of the US National Institutes of Health. The sample sizes for each study are described in the figure legends. All experiments were performed under protocols approved by the Institutional Animal Care and Use Committee at Genentech.

### Generation of *Lrrc15*^*DTRGFP*^ knock-in mouse

Homologous recombination and mouse embryonic stem (ES) cell technology^23–25^ were used to generate a genetically modified mouse strain with *Lrrc15* DTR–GFP knocked-in. A gene-targeting vector was constructed with a 1,704-bp 5′ arm of homology corresponding to GRCm38/mm10 chromosome 16: 30,274,520–30,276,223 and a 1,994-bp arm of 3′ homology arm corresponding to chromosome 16: 30,270,786–30,272,779.  Delete of exon 2 after *ATG* corresponds to chromosome 16: 30,272,780–30,274,516. DTR-EGFP-SV40-FRT-PGK-neo-FRT was inserted immediately after *ATG* of exon 2. The final vector was confirmed by DNA sequencing, linearized and used to target C2 (C57BL/6N) ES cells using standard methods (G418^+^ and ganciclovir^–^ selection)^26^ C57BL/6N C2 ES cells^27^ were electroporated with 20 µg of linearized targeting-vector DNA and cultured under drug selection essentially as previously described^28^. Positive clones were identified using long-range PCR followed by sequence confirmation. Correctly targeted ES cells were subjected to karyotyping. Euploid gene-targeted ES cell clones were treated with Adeno-FLP to remove PGK neomycin, and ES cell clones were tested to identify clones with no copies of the PGK neomycin cassette, and the correct sequence of the targeted allele was verified. The presence of the Y chromosome was verified before microinjection into albino Bl/6N embryos. Germline transmission was obtained after crossing resulting chimeras with C57BL/6N females. Genomic DNA from pups was screened by long-range PCR to verify the desired gene-targeted structure before mouse colony expansion. For genotyping, the following primers were used: 5′-AGGCGAGGCGATTG-3′, 5′-CGATGAGGGCTGAAATGT-3′ and 5′-TGGTCCGTGGATACAGT-3′ amplified 408-bp wild-type and 313-bp knock-in DNA fragments. The following PCR cycle was used: 94 °C for 4 min, (94 °C for 1 min, 55 °C for 30 s, 72 °C for 1 min) for 30 cycles; 72 °C for 10 min; 4 °C ad infinitum.

### Cell lines

The KPR mouse pancreatic adenocarcinoma cell line was generated by the Junttila Group at Genentech from KPR PDAC GEMMs (*Kras*^*LSL.G12D/wt*^*;p16/p19*^*fl/wt*^*;p53*^*LSL.R270H/wt*^;*Pdx1.Cre*)^11^. KPR cells were cultured in RPMI with 10% FBS (Hyclone) plus 2 mmol l^–1^ l-glutamine. All cell lines were tested for *Mycoplasma* contamination by quantitative PCR (Lonza Mycoalert and Stratagene Mycosensor). For all injected tumours, cells were used within the first three passages.

### In vivo tumour studies

For subcutaneous KPR tumours, KPR cells were trypsinized, filtered, counted and resuspended in a 1:1 mixture of Hanks’s buffered saline solution and phenol-red-free Matrigel (Corning) at a concentration of 1 × 10^6^ cells ml^–1^. For all genotypes of mice used, age- and sex-matched 6–12-week-old mice were subcutaneously inoculated in the right unilateral flank with 1 × 10^5^ KPR tumour cells. Flank skin hair was shaved before implantation. Tumour volumes were measured and calculated 2–3 times per week using the following modified ellipsoid formula: ½ × (length × width^2^). Tumours >1,000 mm^3^ were considered progressed and animals were removed from the study. Similarly, animals for which tumours ulcerated greater than 5 mm were removed from the study. For subcutaneous tumour studies in *Lrrc15*^*DTRGFP*^ mice, when tumours reached a volume of 100–200 mm^3^ (about 10 days after inoculation), animals were distributed into treatment groups on the basis of the tumour volume and treatment was initiated.

For orthotopic pancreatic KPR tumours, injection of pancreatic tumour cells into the pancreas of mice was performed as previously described^29^. KPR cells were resuspended in a 1:1 mixture of Hanks’s buffered saline solution and phenol-red-free Matrigel (Corning) at a concentration of either 2 × 10^5^ or 2 × 10^6^ cells ml^–1^. *Dpt*^*CreERT2*^*Tgfbr2*^*fl/fl*^ or *Lrrc15*^*DTR*^ mice were anaesthetized using inhalatory anaesthesia, placed on a heating pad and given eye drop gel. The left flank or abdominal region was shaved and sterilized using ChloraPrep (BD) before making an approximately 1-cm incision with sterile microscissors medial to the splenic silhouette. The underlying muscle layer was incised, and blunt-nose forceps were used to externalize the pancreas and spleen. A prepared 31-gauge needle containing the cell solution was inserted into the tail of the pancreas, and 50 µl of solution containing 1 × 10^5^ cells was slowly injected. The wound was closed using absorbable sutures and wound clips and the mice were allowed to recover. All animals were administered the slow-release analgesic buprenorphine SR LAB at 0.5 mg kg^–1^. Mice were monitored every day after the surgical procedure for signs of infection or distress. For orthotopic tumour studies in *Lrrc15*^*DTRGFP*^ mice, 7 days after implantation, animals were distributed into treatment groups on the basis of the tumour volume and treatment was initiated.

Mice were collected at indicated time points after treatment for analysis or used for tumour growth studies. Sample sizes in the mouse studies were based on the number of mice routinely needed to establish statistical significance based on variability within study groups. Treatment groups were blinded when possible. All animal studies herein were approved by the Genentech Institutional Animal Care and Use Committee.

### Ultrasound imaging of orthotopic pancreatic tumours

For orthotopic pancreatic tumour studies in *Lrrc15*^*DTRGFP*^ mice, tumour volumes were measured by ultrasound imaging. Mice were anaesthetized with 4% sevoflurane (Zoetis) in a warm induction box and positioned on their right side under a continuous flow of 2.5–3% sevoflurane during imaging. Following hair removal, ultrasound coupling gel was placed on the skin, and anatomical B-mode images were acquired on vevo2100 (Fujifilm VisualSonics-) in transverse and longitudinal planes, capturing the maximum tumour cross-sections (MS-550D probe; centre frequency of 40 MHz, axial resolution of 40 µm, lateral resolution of 90 µm and field depth of 12 mm). The pancreatic tumour volume per mouse was analysed using Vevo LAB v.5.5.1 with the following formula for an ellipsoid: volume (mm^3^) = π/6 × length × width × depth.

### In vivo treatments

For TAM-induced *Cre* expression, mice were injected with 2 mg TAM (Sigma, T5648) diluted in sunflower seed oil (Sigma, 88921) for five consecutive days intraperitoneally or were fed chow containing TAM (Envigo, TD.130859). For LRRC15 CAF ablation, mice were intraperitoneally injected with 25 ng g^–1^ of DT (Enzo Life Sciences, BML-G135) twice per week. For CD8-depletion studies, mice were treated with either rat IgG2b isotype control antibody or rat anti-CD8 IgG2b-depleting antibodies (BioXcell, BE0061) at a dose of 10 mg kg^–1^ administered intraperitoneally three times per week. For anti-PDL1 studies, mice were treated with isotype control antibodies or anti-PDL1 (6E11) antibodies (in-house). The first dose was given at 10 mg kg^–1^ followed by 5 mg kg^–1^ thereafter administered intraperitoneally twice per week.

### Mouse tissue digestion, cell isolation and flow cytometry

Tumours were collected, weighed and minced into small pieces. To isolate naive flank skin, hair was shaved, adipose tissue was removed and skin tissue was minced. All tissues were subsequently enzymatically digested using a cocktail of dispase (Life Technologies), collagenase P and DNaseI (Roche) for 45 min at 37 °C to obtain a single-cell suspension. Cells were counted using a Vi-CELL XR (Beckman Coulter). For cytokine staining, cell suspensions were stimulated with eBioscience Cell Stimulation Cocktail plus protein transport inhibitors (00-4975-93) resuspended in RPMI with 10% FBS plus 2 mmol l^–1^ l-glutamine and 2-mercaptoethanol for 2 h at 37 °C. Cells were labelled with the following monoclonal antibodies purchased from BioLegend or BD Biosciences at 4 ºC on ice for 20–30 min, unless otherwise noted. Before cell surface staining with the following fluorescently labelled antibodies, cells were blocked with Fc block (2.4G2; 1:200, 553142). The following surface or intracellular antibodies were used: CD45 (30-F11, 103139); EPCAM (G8.8, 118218); CD31 (390, 561410); PDPN (8.1.1, 127410); CD24 (M1/69, 612832); LRRC15 (M25, in-house); CD90.2 (53-2.1, 565527); CD8 (53-6.7, 612759); PD1 (29F.1A12, 135225); TIM3 (RMT3-23, 119727); LAG3 (C9B7W, 125227); CD39 (Duha59, 143812); IFNγ (XMG1.2, 505846); granzyme B (GB11, 515408); and TNF (MP6-XT22, 506324). Live cells were identified by incubation with calcein blue (Invitrogen, C1429, 1:1,000) after surface staining. For intracellular staining, samples were fixed, permeabilized and stained using a BD Cytofix/Cytoperm Fixation/Permeabilization kit (554714) according to the manufacturer’s instructions. Data were acquired using a Fortessa, Symphony or LSRII (BD Biosciences) flow cytometer and analysed using FlowJo (Tree Star, v.10.7.1), or cells were sorted using a Fusion or Aria (BD Biosciences). instrument Data were processed using Prism GraphPad. Additional information is provided in Supplementary Table 6.

### CAF–CD8 T cell co-culture

For stimulation with plate-bound anti-CD3, 96-well flat-bottomed plates were coated overnight at 4 °C with 10 µg ml^–1^ of anti-CD3 (BioLegend, 100340, clone 145-2C11) and washed once with PBS. Relevant primary CAFs were sorted from digested KPR subcutaneous tumours from DTR^–^ or DTR^+^ mice, and 3 × 10^4^ cells were then added to the anti-CD3-coated well (100 µl). Cells were incubated for 1 h at 37 °C to facilitate attachment. During incubation, mouse CD8^+^ T cells were isolated from single-cell suspension of naive splenocytes by immunomagnetic negative selection using an EasySep Mouse CD8^+^ T cell enrichment kit from Stem Cell (19853) according to the manufacturer’s guidelines. About 6 × 10^4^ purified CD8^+^ T cells were added to the wells in the presence of soluble 0.50 µg ml^–1^ anti-CD28 (BioLegend, 102115, clone 37.51) (100 µl). On the day of analysis, medium was replaced and cells were cultured with 1× Cell Stimulation Cocktail (eBioscience 500× Cell Stimulation Cocktail plus protein transport inhibitors, 00-4975) and 55 µM 2-mercaptoethanol for 4 h at 37 °C. Cells were collected, filtered and stained for surface markers. Following surface staining, cells were fixed and permeabilized with Intracellular Fixation and Permeabilization Buffer Set according to the manufacturer’s guidelines before staining for intracellular cytokines. Cells were then analysed by flow cytometry.

### Immunofluorescence and image analysis of mouse tumours

Tumours were fixed overnight in 4% paraformaldehyde and embedded in optimal cutting temperature medium (Sakura Finetek) and frozen for storage at −80 °C. Sections (8–12 μm thick) were cryosectioned and stained. For staining, slides were blocked and permeabilized with normal mouse serum (1:50), mouse Fc block (1:100) and 0.3% Triton-X diluted in PBS for 30 min at room temperature. Tissue sections were incubated with primary antibodies for 1 h at room temperature or overnight at 4 °C. After washing, secondary antibodies were added for 1 h at room temperature. To counterstain, slides were rinsed and incubated with DAPI (ThermoFisher, D1306) at 300 nM in PBS for 5 min at room temperature. Details of the antibodies used can be found in Supplementary Table 6. Slides were rinsed several times in PBS, excess buffer drained and sections were mounted in Vectashield (H-1000). Images were acquired with a Nikon A1R confocal microscope equipped with a Plan apo lambda NA 0.75 ×20 lens. Lasers were set at excitation at 488 nm, 561 nm and 640 nm, and a perfect focus module. NIS Elements acquisition software was used with a digital zoom of 2 for full tissue section imaging or 7 for details, and stitching of single plane images was performed. Estimations of CD8 T cell–LRRC15 CAF interaction rates were compiled between T cells and CAFs observed among total T cells in a 500 × 500 × 10 µm^3^ section of tissue across 3 different tumours.

### *Lrrc15* in situ hybridization

Tissues for in situ hybridization were formalin-fixed and paraffin-embedded. Mouse LRRC15 in situ hybridization was performed using an ACD probe (Advanced Cell Diagnostics, 467838 with 120 min hybridization. ER2 retrieval (Leica) at 95 °C for 15 min and RNAscope 2.5 LS Protease III digestion (ACD) was performed on a Leica Bond autostainer. RNAscope 2.5 LS Reagent Kit Red (ACD) was used for detection.

### Mouse scRNA-seq and cell hashing

Mouse scRNA-seq and cell hashing with unique barcoded antibodies (BioLegend) were processed using Chromium Single Cell Gene Expression 3′ v3 Library and a Gel Bead kit following the manufacturer’s instructions (10x Genomics, PN-1000075). Cells were counted and checked for viability using a Vi-CELL XR cell counter (Beckman Coulter), and then injected into microfluidic chips to form gel beads-in-emulsion in a 10x Chromium instrument. Reverse transcription was performed on the gel beads-in-emulsion, and products were purified and amplified. DNA from antibody-derived tags was separated from cDNA based on size selection using SPRIselect beads (Beckman Coulter, B23318). Expression libraries and antibody-derived tag libraries were generated and profiled using a Bioanalyzer High Sensitivity DNA kit (Agilent Technologies, 5067-4626) and quantified with a Kapa Library Quantification kit (Roche, 07960255001). All libraries were sequenced using HiSeq4000 and NovaSeq (Illumina)

### Mouse scRNA-seq data processing

#### Initial data processing

scRNA-seq data for each library from each cell type were processed with CellRanger count (CellRanger 3.1, 10x Genomics) with a custom reference based on the mouse reference genome GRCm38 and GENCODE gene models. Counts of barcode antibodies to label individual replicates were processed using DemuxEM with default parameters to assign individual sample labels^30^. Cells identified as doublets or HTO-negative cells were excluded from the analysis. For gene expression counts, individual samples were merged into one expression matrix and analysed using the package Seurat. Cells with fewer than 300 expressed genes or more than 5% mitochondrial counts were removed. Transcript counts were log-normalized (Seurat, NormalizeData), and the top 2,000 most variable genes were selected using variance stabilizing transformation (FindVariableFeatures), followed by data scaling (ScaleData). PCA was then performed on this gene space (RunPCA). Clustering was carried out on the basis of the shared nearest neighbour between cells (FindNeighbors, 30 PCs) and graph-based clustering (30 PCs, resolution of 0.1 for *Lrrc15* depletion, 0.5 for *Tgfbr2* KO experiments). We calculated markers for individual clusters using the FindMarkers function in Seurat (Wilcoxon’s rank sum test, Benjamini–Hochberg adjustment for multiple testing). To visualize gene expression levels for individual clusters, we calculated the average gene expression in each cluster and calculated a *z*-score value on a by-gene basis.

#### Filtering of cells

For the *Dpt*^*IresCreERT2*^*Tgfbr2*^*fl/fl*^ experiments, cells in the resulting Seurat object from the initial data-processing step were further filtered based on the expression of known cell-type markers. Only fibroblast cells from clusters 0, 2, 3, 4, 5 and 8 expressing *Lum* and *Dcn*, but not *Pecam1* (endothelial cells), *Ptprc* (immune cells) or *Rgs5* (pericytes), were retained for subsequent analyses. We then performed dimensionality reduction and clustering as described above and removed remaining minor contaminant non-fibroblast cells. The final dimensionality reduction and clustering were performed using 30 PCs and a clustering resolution of 0.3.

For the *Lrrc15*^*DTRGFP*^ depletion experiments, fibroblasts cells (clusters 0, 1, 2 and 5) in the resulting Seurat object from the initial data-processing step were isolated by excluding clusters expressing *Pecam1* (endothelial cells), *Ptprc* (immune cells)*, Rgs5* (pericytes), *Krt18* (epithelial) or *Myl1* (smooth muscle cells). We then performed dimensionality reduction and clustering as described above and removed remaining minor contaminant non-fibroblast cells. The final dimensionality reduction and clustering were performed using 30 PCs and a clustering resolution of 0.2.

#### Scoring of cells for gene expression programmes

Cells were scored for gene expression programmes using the addModuleScore function in Seurat and a gene set of interest as input. PDAC mouse GEMM programmes were derived as follows. We used genes with at least 0.6 average log fold-change upregulation in TGFβ CAFs (cluster 2) from our previous study^1^ as marker genes for these conditions. To identify a normal tissue fibroblast (clusters 3 and 4) gene set, we identified the top 20 markers for clusters 3 and 4 compared these to all other cells in the dataset using the FindMarkers function in Seurat.

#### Population frequency analysis

To assess differences in abundance of cells from specific clusters between conditions, we used the R package speckle (https://github.com/Oshlack/speckle), which is designed for finding significant differences in cell-type proportions. In brief, speckle calculates the fraction of cells assigned to a particular cluster in each biological replicate, performs a variance stabilizing transformation on the proportions and determines whether the cell-type proportions are significant between different groups. Given that we only compared two groups in all our experiments, *t*-test was used by speckle to calculate *P* values, which were adjusted for multiple testing using Benjamini–Hochberg correction.

#### Pathway enrichment analysis

We used PROGENy^17^ to infer pathway activity from our single-cell gene expression data as previously described^10^ and following the single-cell tutorial provided by the authors (https://saezlab.github.io/progeny/articles/ProgenySingleCell.html). We matched progeny scores with either clusters or experimental time point/condition and summarized the data by population.

### *Lrrc15* gene expression in mouse tissues

Normalized fragments per kilobase of sequence per million mapped read values were retrieved from Supplementary Table 6 in ref. ^12^. Data were log-transformed and expression levels of *Lrrc15* and *Gapdh* were visualized by tissue.

### TCGA data analysis

Batch-corrected normalized TCGA Pan-Cancer mRNA data were obtained from UCSC Xenabrowser (https://xenabrowser.net/) (*N* = 11,060). Samples containing NA expression values were removed. We additionally filtered the data to only contain samples from primary solid tumours (sample code 01; *N* = 9,702). Survival data were obtained from Table S1 in ref. ^31^ and linked to the Pan-Cancer dataset using the unique TCGA participant barcode. Indications with fewer than 80 patients were excluded from the analysis (final dataset: *N* = 9,195 patients). *TGFB* CAF levels were inferred by calculating the average expression of our previously published marker signature^1^ within a sample after *z*-score transformation of each gene across samples. Association with survival across TCGA data was determined with multivariate Cox regression and TCGA indication as a covariate, as well as univariate Cox regression analysis within each indication. The hazard ratio was defined as the change in the risk of death if the TGFβ CAF score increased by one unit.

### Human tumour digestion and stromal cell RNA-seq analysis

#### Tumour collection

Tumour samples for the Immunoprofiler Initiative (IPI) were transported from various cancer operating rooms and from outpatient clinics. All patients provided consent to the University of California, San Francisco (UCSF) IPI clinical coordinator group for tissue collection under a UCSF protocol approved by the institutional review board (UCSF IRB 20-31740). Samples were obtained after surgical excision with biopsies taken by pathology assistants to confirm the presence of tumour cells. Patients were selected without regard to previous treatment. Freshly resected samples were placed in ice-cold PBS or Leibovitz’s L-15 medium in a 50-ml conical tube and immediately transported to the laboratory for sample labelling and processing. Samples were used for either whole-tissue digestion into a single-cell suspension or a part of the tissue was sliced and preserved for imaging analysis.

#### Cell sorting, library preparation and sequencing

Cell sorting, library preparation, sequencing and bioinformatics data processing were performed as previously described^32^.

#### Computational analysis of sorted stromal RNA-seq

From the log-transformed matrix of normalized gene × cell expression values, we identified the 2,500 most variable genes based on their interquartile range and performed PCA in the space of these genes. We then used the 10 most strongly positively and negatively loading genes of PC1–PC6 for hierarchical clustering of samples and genes (complete linkage and Euclidean distance). The cluster dendrogram was split into *k* = 6 clusters based on tree height. We interpreted clusters of samples with high expression of *EPCAM*, *KRT8 and KRT18* as epithelial-driven and *TYROBP* and *CSF3R* as myeloid-driven and excluded these samples from our subsequent analysis. Next, we performed PCA on the remaining samples. The loadings of the resulting PC space were then used to project the epithelial- and myeloid-driven samples onto PC1 and PC2.  Similarly, microdissected bulk RNA-seq samples from patients with PDAC as provided in ref. ^33^ were obtained from the Gene Expression Omnibus database (identifier GSE93326) and projected onto PC1 and PC2.

### Reporting summary

Further information on research design is available in the Nature Research Reporting Summary linked to this article.

## Online content

Any methods, additional references, Nature Research reporting summaries, source data, extended data, supplementary information, acknowledgements, peer review information; details of author contributions and competing interests; and statements of data and code availability are available at 10.1038/s41586-022-05272-1.

## Supplementary information

Reporting Summary

Supplementary Table 1 Markers of all cells for the *Dpt*^I^^*resCreERT2*^*Tgfbr2*^*fl/fl*^ scRNA-seq experiment.

Supplementary Table 2 Markers of fibroblasts for the *Dpt*^*IresCreERT2*^*Tgfbr*2^fl/fl^ scRNA-seq experiment.

Supplementary Table 3 Patient information for the IPI cohort.

Supplementary Table 4 Markers for all cells for the *Lrrc15*^*DTR*^ kinetic scRNA-seq experiment.

Supplementary Table 5 Markers for fibroblasts for the *Lrrc15*^DTR^ kinetic scRNA-seq experiment.

Supplementary Table 6 Information for antibodies used in this manuscript.

## Data Availability

Raw and processed scRNA-seq datasets are available from the ArrayExpress repository under the accession numbers E-MTAB-12028 and E-MTAB-12036. Human stromal cell bulk RNA-seq data are available in the European Genome–Phenome Archive database under accession EGAD00001009176. Source data are provided with this paper.

## Source data

Source Data Fig. 1
Source Data Fig. 2
Source Data Fig. 3
Source Data Fig. 4
Source Data Extended Data Fig. 1
Source Data Extended Data Fig. 2
Source Data Extended Data Fig. 3
Source Data Extended Data Fig. 4
Source Data Extended Data Fig. 5
Source Data Extended Data Fig. 6
Source Data Extended Data Fig. 7

## Code availability

No new algorithms were developed for this manuscript. All code generated for analysis is available from the authors upon request.

## References

[1] Dominguez CX (2020). Single-cell RNA sequencing reveals stromal evolution into LRRC15^+^ myofibroblasts as a determinant of patient response to cancer immunotherapy. Cancer Discov..

[2] Kieffer Y (2020). Single-cell analysis reveals fibroblast clusters linked to immunotherapy resistance in cancer. Cancer Discov..

[3] Bartoschek M (2018). Spatially and functionally distinct subclasses of breast cancer-associated fibroblasts revealed by single cell RNA sequencing. Nat. Commun..

[4] Sahai E (2020). A framework for advancing our understanding of cancer-associated fibroblasts. Nat. Rev. Cancer.

[5] Biffi G, Tuveson DA (2021). Diversity and Biology of cancer-associated fibroblasts. Physiol. Rev..

[6] Baker AT, Abuwarwar MH, Poly L, Wilkins S, Fletcher AL (2021). Cancer-associated fibroblasts and T cells: from mechanisms to outcomes. J. Immunol..

[7] Mariathasan S (2018). TGFβ attenuates tumour response to PD-L1 blockade by contributing to exclusion of T cells. Nature.

[8] Chakravarthy A, Khan L, Bensler NP, Bose P, Carvalho DDD (2018). TGF-β-associated extracellular matrix genes link cancer-associated fibroblasts to immune evasion and immunotherapy failure. Nat. Commun..

[9] Biffi G (2019). IL1-induced JAK/STAT signaling is antagonized by TGFβ to shape CAF heterogeneity in pancreatic ductal adenocarcinoma. Cancer Discov..

[10] Buechler MB (2021). Cross-tissue organization of the fibroblast lineage. Nature.

[11] Chung W-J (2017). Kras mutant genetically engineered mouse models of human cancers are genomically heterogeneous. Proc. Natl Acad. Sci. USA.

[12] Li B (2017). A comprehensive mouse transcriptomic BodyMap across 17 tissues by RNA-seq. Sci Rep..

[13] Purcell JW (2018). LRRC15 is a novel mesenchymal protein and stromal target for antibody–drug conjugates. Cancer Res..

[14] Özdemir BC (2014). Depletion of carcinoma-associated fibroblasts and fibrosis induces immunosuppression and accelerates pancreas cancer with reduced survival.. Cancer Cell.

[15] Kraman M (2010). Suppression of antitumor immunity by stromal cells expressing fibroblast activation protein-α. Science.

[16] Kapoor VN (2021). Gremlin 1^+^ fibroblastic niche maintains dendritic cell homeostasis in lymphoid tissues. Nat. Immunol..

[17] Schubert M (2018). Perturbation-response genes reveal signaling footprints in cancer gene expression. Nat. Commun..

[18] Wherry EJ, Kurachi M (2015). Molecular and cellular insights into T cell exhaustion. Nat. Rev. Immunol..

[19] Gupta PK (2015). CD39 expression identifies terminally exhausted CD8^+^ T cells. PLoS Pathog..

[20] Feig C (2013). Targeting CXCL12 from FAP-expressing carcinoma-associated fibroblasts synergizes with anti-PD-L1 immunotherapy in pancreatic cancer. Proc. Natl Acad. Sci. USA.

[21] Cremasco V (2014). B cell homeostasis and follicle confines are governed by fibroblastic reticular cells. Nat. Immunol..

[22] Derynck R, Turley SJ, Akhurst RJ (2021). TGFβ biology in cancer progression and immunotherapy. Nat. Rev. Clin. Oncol..

[23] Kuehn MR, Bradley A, Robertson EJ, Evans MJ (1987). A potential animal model for Lesch–Nyhan syndrome through introduction of *HPRT* mutations into mice. Nature.

[24] Smithies O, Gregg RG, Boggs SS, Koralewski MA, Kucherlapati RS (1985). Insertion of DNA sequences into the human chromosomal β-globin locus by homologous recombination. Nature.

[25] Thomas KR, Folger KR, Capecchi MR (1986). High frequency targeting of genes to specific sites in the mammalian genome. Cell.

[26] Newman RJ, Roose-Girma M, Warming S (2015). Efficient conditional knockout targeting vector construction using co-selection BAC recombineering (CoSBR). Nucleic Acids Res..

[27] Gertsenstein M (2010). Efficient generation of germ line transmitting chimeras from C57BL/6N ES cells by aggregation with outbred host embryos. PLoS ONE.

[28] Hughes, E. D. & Saunders, T. L. in *Advanced Protocols for Animal Transgenesis: An ISTT Manual* (eds Pease, S. & Saunders, T. L.) 291–325 (Springer, 2011).

[29] Aiello, N. M., Rhim, A. D. & Stanger, B. Z. Orthotopic injection of pancreatic cancer cells. *Cold Spring Harb. Protoc.*10.1101/pdb.prot078360 (2016). 10.1101/pdb.prot078360 26729902

[30] Gaublomme JT (2019). Nuclei multiplexing with barcoded antibodies for single-nucleus genomics. Nat. Commun..

[31] Thorsson V (2018). The immune landscape of cancer. Immunity.

[32] Combes AJ (2022). Discovering dominant tumor immune archetypes in a pan-cancer census. Cell.

[33] Maurer C (2019). Experimental microdissection enables functional harmonisation of pancreatic cancer subtypes. Gut.

