# Supplementary material for: LRRC15+ myofibroblasts dictate the stromal setpoint to suppress tumour immunity
Source: Nature. 2022 Sep 28;611(7934):148–54. doi: 10.1038/s41586-022-05272-1 (PMC9630141; doi:10.1038/s41586-022-05272-1)
Supplement: Supplementary file 1 — Reporting Summary [file 41586_2022_5272_MOESM1_ESM.pdf]

nature portfolio

Corresponding author(s): Dr. Shannon Turley; Dr. Soren Muller

Last updated by author(s): Aug 8, 2022

Reporting Summary

Nature Portfolio wishes to improve the reproducibility of the work that we publish. This form provides structure for consistency and transparency in reporting. For further information on Nature Portfolio policies, see our Editorial Policies and the Editorial Policy Checklist.

Statistics

For all statistical analyses, confirm that the following items are present in the figure legend, table legend, main text, or Methods section.

n/a	Confirmed	
☐	☒	The exact sample size (n) for each experimental group/condition, given as a discrete number and unit of measurement
☐	☒	A statement on whether measurements were taken from distinct samples or whether the same sample was measured repeatedly
☐	☒	The statistical test(s) used AND whether they are one- or two-sided<br>*Only common tests should be described solely by name; describe more complex techniques in the Methods section.*
☒	☐	A description of all covariates tested
☐	☒	A description of any assumptions or corrections, such as tests of normality and adjustment for multiple comparisons
☐	☒	A full description of the statistical parameters including central tendency (e.g. means) or other basic estimates (e.g. regression coefficient) AND variation (e.g. standard deviation) or associated estimates of uncertainty (e.g. confidence intervals)
☐	☒	For null hypothesis testing, the test statistic (e.g. F, t, r) with confidence intervals, effect sizes, degrees of freedom and P value noted<br>*Give P values as exact values whenever suitable.*
☒	☐	For Bayesian analysis, information on the choice of priors and Markov chain Monte Carlo settings
☒	☐	For hierarchical and complex designs, identification of the appropriate level for tests and full reporting of outcomes
☒	☐	Estimates of effect sizes (e.g. Cohen's d, Pearson's r), indicating how they were calculated

Our web collection on statistics for biologists contains articles on many of the points above.

Software and code

Policy information about availability of computer code

Data collection	No software was used for data collection
Data analysis	Prism, GraphPad (v9)<br>Flowjo (v10.7.1)<br>R (3.6.1)<br>Seurat (3.1.4)<br>Progeny (1.13.0)<br>Pheatmap (1.0.12)<br>speckle (0.0.1)<br>Vevo LAB v5.5.1<br>NIS Elements AR analysis 5.21.03 64-bit

For manuscripts utilizing custom algorithms or software that are central to the research but not yet described in published literature, software must be made available to editors and reviewers. We strongly encourage code deposition in a community repository (e.g. GitHub). See the Nature Portfolio guidelines for submitting code & software for further information.

Data

Policy information about availability of data

All manuscripts must include a data availability statement. This statement should provide the following information, where applicable:

- Accession codes, unique identifiers, or web links for publicly available datasets
- A description of any restrictions on data availability
- For clinical datasets or third party data, please ensure that the statement adheres to our policy

Raw and processed scRNA-seq datasets are available from the ArrayExpress repository under the accession numbers E-MTAB-12028 and E-MTAB-12036. Human stromal cell bulk RNA-seq data are available in the EGA database under accession EGAD00001009176.

Field-specific reporting

Please select the one below that is the best fit for your research. If you are not sure, read the appropriate sections before making your selection.

☒ Life sciences ☐ Behavioural & social sciences ☐ Ecological, evolutionary & environmental sciences

For a reference copy of the document with all sections, see nature.com/documents/nr-reporting-summary-flat.pdf

Life sciences study design

All studies must disclose on these points even when the disclosure is negative.

Sample size	No statistical methods were used to pre-determine sample size. Sample sizes were based on preliminary experimentation and we designed our experiments to achieve a minimum of n=3, but mostly n=5 or greater, mice (or samples) per group or condition. This enabled us to carry out biologically significant experiments with reproducible results.
Data exclusions	No data was excluded from analyses
Replication	All experiments were performed at least two independent times under identical conditions, unless otherwise stated (see Figure legends).
Randomization	For animal studies, all mice used were sex and age matched. Mice were grouped based on genotype and randomization was not required for the experiments performed. For non-animal experiments, randomization was not required as studies in human subjects were focused on fibroblast heterogeneity across all samples and patients as a whole.
Blinding	In all animal studies, blinding was not necessary and animals were grouped based on genotype

Reporting for specific materials, systems and methods

We require information from authors about some types of materials, experimental systems and methods used in many studies. Here, indicate whether each material, system or method listed is relevant to your study. If you are not sure if a list item applies to your research, read the appropriate section before selecting a response.

Materials & experimental systems

n/a	Involved in the study	
☐	☒	Antibodies
☒	☐	Eukaryotic cell lines
☒	☐	Palaeontology and archaeology
☐	☒	Animals and other organisms
☐	☒	Human research participants
☒	☐	Clinical data
☒	☐	Dual use research of concern

Methods

n/a	Involved in the study	
☒	☐	ChIP-seq
☐	☒	Flow cytometry
☒	☐	MRI-based neuroimaging

Antibodies

Antibodies used	See supplementary table 6 for information on all antibodies used in these studies, including supplier name, catalog number, clone name, and dilution used.
Validation	Antibodies used have been validated by vendors and reference links for this validation for each antibody are provided in Supplementary Table 6. The markers used are standard flow cytometry immunological markers.

Animals and other organisms

Policy information about studies involving animals; ARRIVE guidelines recommended for reporting animal research

Laboratory animals	DptIresCreERT2 mice and Lrrc15DTR-GFP mice were designed, generated and bred at Genentech. Tgfβr2fl/fl mice (012603) were obtained from Jackson Laboratory. Age and sex-matched mice (male and female) 6–12 weeks old were used for all studies. Mice were maintained under specific pathogen-free conditions using the guidelines of the US National Institutes of health.
Wild animals	The study did not involve the use of wild animals
Field-collected samples	The study did not involve samples collected from the field.
Ethics oversight	All experiments were performed under protocols approved by the Institutional Animal Care and Use Committee at Genentech.

Note that full information on the approval of the study protocol must also be provided in the manuscript.

Human research participants

Policy information about studies involving human research participants

Population characteristics	Patient information is given in Suppl. Table S3
Recruitment	Tumor samples for the Immunoprofiler was transported from various UCSF cancer operating rooms (ORs) as well as from outpatient clinics.
Ethics oversight	All patients consented by the UCSF IPI clinical coordinator group for tissue collection under a UCSF IRB approved protocol (UCSF IRB# 20-31740).

Note that full information on the approval of the study protocol must also be provided in the manuscript.

Flow Cytometry

Plots

Confirm that:

- ☒ The axis labels state the marker and fluorochrome used (e.g. CD4-FITC).
- ☒ The axis scales are clearly visible. Include numbers along axes only for bottom left plot of group (a 'group' is an analysis of identical markers).
- ☒ All plots are contour plots with outliers or pseudocolor plots.
- ☒ A numerical value for number of cells or percentage (with statistics) is provided.

Methodology

Sample preparation	Mouse tumors were collected, weighed and minced into small pieces. To isolate naive flank skin, hair was shaved, adipose tissue was removed and skin tissue was minced. All tissues were subsequently enzymatically digested using a cocktail of dispase (Life Technologies, Carlsbad, CA), collagenase P and DNaseI (Roche, Penzberg, Germany) for 45 min at 37°C, to obtain a single cell suspension. Cells were counted using a Vi-CELL XR (Beckman Coulter, Brea, CA).
Instrument	Data were acquired on a Fortessa, Symphony or LSRII (BD Biosciences) or cells were sorted on a Fusion or Aria (BD Biosciences).
Software	FlowJo (Tree Star, v10.7.1)
Cell population abundance	Cells were sorted at >=90% purity as assess on FACS Aria or Fusion.
Gating strategy	For all samples, cells were first gated on singlets and then by the viability marker to gate live cells. For cell subsetting, tumor cells, marked as CD24+CD45-, were first excluded followed by gating on CD45+ cells for immune cells or CD24-CD45- cells for stromal cells. Subsequent lineage gating was used for subsets within each compartment.

☒ Tick this box to confirm that a figure exemplifying the gating strategy is provided in the Supplementary Information.
